# Infections with *Staphylococcus* spp. in Children Undergoing Anticancer Therapy or Haematopoietic Cell Transplantation: A Nationwide Multicentre Study

**DOI:** 10.3390/jcm14155200

**Published:** 2025-07-22

**Authors:** Anna Jabłońska, Monika Richert-Przygońska, Kamila Jaremek, Krzysztof Czyżewski, Wanda Badowska, Walentyna Balwierz, Ewa Bień, Tomasz Brzeski, Radosław Chaber, Wojciech Czogała, Bożenna Dembowska-Bagińska, Katarzyna Derwich, Katarzyna Drabko, Katarzyna Dzierżanowska-Fangrat, Jowita Frączkiewicz, Agnieszka Gietka, Jolanta Goździk, Olga Gryniewicz-Kwiatkowska, Łukasz Hutnik, Ninela Irga-Jaworska, Krzysztof Kałwak, Grażyna Karolczyk, Aleksandra Królak, Pawel Łaguna, Katarzyna Machnik, Hanna Mańko-Glińska, Agnieszka Mizia-Malarz, Wojciech Młynarski, Jakub Musiał, Katarzyna Mycko, Tomasz Ociepa, Sonia Pająk, Jarosław Peregud-Pogorzelski, Filip Pierlejewski, Marcin Płonowski, Małgorzata Salamonowicz-Bodzioch, Małgorzata Sawicka-Żukowska, Katarzyna Semczuk, Katarzyna Skowron-Kandzia, Weronika Stolpa, Tomasz Szczepański, Anna Szmydki-Baran, Renata Tomaszewska, Tomasz Urasiński, Agnieszka Urbanek-Dądela, Justyna Urbańska-Rakus, Paweł Wawryków, Olga Zając-Spychała, Patrycja Zalas-Więcek, Agnieszka Zaucha-Prażmo, Joanna Zawitkowska, Iwona Żak, Jan Styczyński

**Affiliations:** 1 Doctoral School of Medical and Health Sciences, Ludwik Rydygier Collegium Medicum, Nicolaus Copernicus University, 85-067 Bydgoszcz, Poland; 2 Department of Paediatric Haematology and Oncology, Ludwik Rydygier Collegium Medicum, Nicolaus Copernicus University, 85-094 Bydgoszcz, Poland; monika_richert@yahoo.com (M.R.-P.); kjaremem@gmail.com (K.J.); k.czyzewski@cm.umk.pl (K.C.); jstyczynski@cm.umk.pl (J.S.); 3 Department of Paediatric Oncology, Haematology and Transplantology, University of Medical Sciences, 61-701 Poznan, Poland; kderwich@ump.edu.pl (K.D.); olga_zajac@wp.pl (O.Z.-S.); 4 Department of Clinical Paediatrics, University of Warmia and Mazury, Clinical Division of Paediatric Oncology and Haematology, Regional Specialised Children’s Hospital, 10-561 Olsztyn, Poland; bwan@o2.pl (W.B.); tomasz.brzeski@uwm.edu.pl (T.B.); haniam@autograf.pl (H.M.-G.); katarzynamycko@tlen.pl (K.M.); 5 Department of Paediatric Oncology and Haematology, Institute of Paediatrics, Jagiellonian University Medical College, 31-008 Krakow, Poland; balwierz@mp.pl (W.B.); wojciech.czogala@uj.edu.pl (W.C.); 6 Department of Paediatrics, Haematology and Oncology, Medical University, 80-210 Gdansk, Poland; ebien@gumed.edu.pl (E.B.); nirga@gumed.edu.pl (N.I.-J.); 7 Department of Paediatric Oncohaematology, Medical Faculty University of Rzeszow, Clinical Provincial Hospital No. 2, 35-301 Rzeszow, Poland; rchaber@wp.pl (R.C.);; 8 Department of Oncology, Children’s Memorial Health Institute, 04-730 Warsaw, Poland; b.dembowska@ipczd.pl (B.D.-B.); a.gietka@ipczd.pl (A.G.); o.gryniewicz@ipczd.pl (O.G.-K.); 9 Department of Paediatric Haematology, Oncology and Transplantology, Medical University of Lublin, 20-059 Lublin, Polandagnieszkazauchaprazmo@umlub.pl (A.Z.-P.); joannazawitkowska@umlub.pl (J.Z.); 10 Department of Clinical Microbiology and Immunology, Children’s Memorial Health Institute, 04-730 Warsaw, Poland; k.fangrat@ipczd.pl (K.D.-F.); k.semczuk@ipczd.pl (K.S.); 11 Department of Paediatric Haematology, Oncology and BMT, Wroclaw Medical University, 50-367 Wrocław, Poland; jowitafr@gmail.com (J.F.); krzysztof.kalwak@gmail.com (K.K.); msalamonowicz@poczta.onet.pl (M.S.-B.); 12 Stem Cell Transplant Center, University Children’s Hospital, Department of Clinical Immunology and Transplantology, Jagiellonian University Collegium Medicum, 31-008 Krakow, Poland; jgozdzik@cm-uj.krakow.pl; 13 Department of Oncology, Paediatric Haematology, Clinical Transplantation and Paediatrics, Medical University of Warsaw, 02-091 Warsaw, Poland; lukasz.hutnik@gmail.com (Ł.H.); pawel.laguna@wum.edu.pl (P.Ł.); aszmydki@tlen.pl (A.S.-B.); 14 Division of Paediatric Haematology and Oncology, Children Hospital, 25-736 Kielce, Poland; grazyna.karolczyk@wszzkielce.pl (G.K.); agnieszka.urbanek-dadela@wszzkielce.pl (A.U.-D.); 15 Department of Paediatrics and Haemato-Oncology, Pomeranian Medical University, 70-204 Szczecin, Poland; aleksandra.krolak@pum.edu.pl (A.K.); tomasz.ociepa@pum.edu.pl (T.O.); tomasz.urasinski@pum.edu.pl (T.U.); 16 Division of Paediatric Haematology and Oncology, Chorzow City Hospital, 41-500 Chorzow, Poland; katmachnik@gmail.com (K.M.); s.pajak94@gmail.com (S.P.); justyna.urbanska.rakus@gmail.com (J.U.-R.); 17 Division of Paediatric Oncology, Haematology and Chemotherapy, Department of Paediatrics, Silesian Medical University, 40-055 Katowice, Poland; a.mizia@hotmail.com (A.M.-M.); kasia.skowron@gmail.com (K.S.-K.); wera1pl@poczta.onet.pl (W.S.); 18 Department of Paediatrics, Haematology and Oncology, Medical University, 90-419 Lodz, Poland; wojciech.mlynarski@umed.lodz.pl (W.M.); filip.pierlejewski@umed.lodz.pl (F.P.); 19 Department of Paediatrics, Paediatric Oncology and Immunology, Pomeranian Medical University, 70-204 Szczecin, Poland; jwperegud@gmail.com (J.P.-P.); pawel.wawrykow@gmail.com (P.W.); 20 Department of Paediatric Oncology and Haematology, Medical University, 15-089 Bialystok, Poland; mar26@mp.pl (M.P.); malgorzata.sawicka-zukowska@umb.edu.pl (M.S.-Ż.); 21 Department of Paediatric Haematology and Oncology, Silesian Medical University, 41-808 Zabrze, Poland; szczep57@poczta.onet.pl (T.S.); tomaszewskar@gmail.com (R.T.); 22 Microbiology Department, Ludwik Rydygier Collegium Medicum, Nicolaus Copernicus University, 85-094 Bydgoszcz, Poland; p.zalas@cm.umk.pl; 23 Department of Microbiology, University Children’s Hospital, 30-663 Krakow, Poland; izak@usdk.pl

**Keywords:** oncology, paediatric malignancy, haematopoietic cell transplantation, *Staphylococcus*, infection, bacteraemia, leukaemia

## Abstract

**Background**: *Staphylococcus* spp. represent the most prevalent Gram-positive organisms in children with malignancies or undergoing haematopoietic cell transplantation (HCT), contributing to significant morbidity and mortality. This study aimed to assess the epidemiology, risk factors, treatment strategies, and outcomes of staphylococcal infections (SIs) in paediatric haemato-oncology (PHO) and HCT patients in Poland over a 12-year period. **Methods**: A retrospective, multicentre study was conducted across 17 paediatric oncology centres in Poland. The clinical and microbiological data of patients under the age of 18, diagnosed with malignancies or post-HCT, were analysed for confirmed SI between 2012 and 2023. The variables assessed included demographics, underlying conditions, infection type and source, antimicrobial susceptibility, treatment, and 30-day infection-free survival. **Results**: Among 1725 patients with SI, 1433 were PHO and 292 were HCT patients. The cumulative incidence of SI was 12.7% in PHO and 14.3% in HCT patients (*p* = 0.008). The 30-day survival rate was significantly higher in PHO compared to HCT patients (98.4% vs. 93.2%, *p* < 0.001). Most deaths were caused by *S. epidermidis*, *S. haemolyticus*, and *S. hominis*, predominantly involving methicillin-resistant coagulase-negative *Staphylococci (MRCNS)*. Multivariate Cox regression identified undergoing HCT (HR = 3.0, 95% CI: 1.6–5.6, *p* < 0.001) and treatment of infection > 10 days (HR = 2.0, 95% CI: 1.1–3.6, *p* = 0.019) as independent risk factors for mortality. **Conclusions**: Staphylococcal infections pose a significant challenge in paediatric oncology and transplant populations. Optimising prevention, diagnostics, and antimicrobial therapy is crucial for improving outcomes in these high-risk groups.

## Simple Summary

Staphylococcal infections (SIs) are among the most frequent contributors to morbidity and mortality in paediatric oncology (PHO) and haematopoietic cell transplantation (HCT) patients. This study, performed over 12 years across 17 centres in Poland, evaluated the incidence, risk factors, and outcomes of SI in 1725 paediatric patients. The cumulative incidence of infection was significantly higher in HCT recipients compared to PHO patients, with recurrent infections being more prevalent in the former group. The first episode of infection occurred much earlier in HCT patients. Infection-related mortality was higher in the HCT cohort, and methicillin-resistant coagulase-negative *Staphylococci* (MRCNS) were the predominant pathogen in fatal cases. The multivariate analysis revealed that undergoing HCT or requiring extended treatment were independent risk factors for mortality. These findings underscore the need for enhanced infection prevention strategies and early, tailored antimicrobial therapy in this vulnerable paediatric population.

## 1. Introduction

Children with malignancies or undergoing haematopoietic cell transplantation or other cellular therapies represent a group at high risk of developing infectious complications. Infections have a negative impact on treatment outcomes and can contribute to additional complications, making them a major cause of death in paediatric oncology units. An important goal in oncology is to optimise supportive therapy, including the prevention and treatment of infections. Several factors make children with malignancies more susceptible to infections, such as the disruption of natural barriers, presence of central venous catheters, immunosuppression, intensive chemotherapy, and neutropenia [1]. In recent years, an increase in Gram-positive bacterial infections has been observed in patients with malignancies, particularly driven by *Staphylococcus aureus* and coagulase-negative *Staphylococcus* (CoNS), alongside a decrease in Gram-negative bacteraemia [2,3].

*Staphylococcus* spp. are responsible for a variety of infections. *S. aureus* commonly causes bloodstream infections, and a significant proportion of these infections are associated with vascular catheter use [4]. *S. epidermidis* is also an important aetiological agent of sepsis in children with leukaemia and lymphoma [5]. In addition, *Staphylococcus* spp. can cause skin and soft tissue infections, such as abscesses and folliculitis, and respiratory tract infections, including pneumonia [6,7]. However, it should be noted that most available studies have focused on bloodstream infections, and data on other types of infections are limited. Similarly, although several studies have investigated the epidemiology and risk factors of staphylococcal infections (SIs) in adults, data on paediatric populations are limited. A major concern in oncology is methicillin-resistant *Staphylococcus aureus* (MRSA) infection in colonised patients. MRSA is a pathogen in which prior colonisation increases the risk of invasive infection in paediatric oncology patients, which can lead to rapidly progressive and potentially fatal infections [8]. A better understanding of the risk factors, infection-related outcomes, and survival of patients in this group is essential for optimising clinical management strategies.

The aim of this study was to analyse the incidence and outcome of staphylococcal infections in patients treated in paediatric haemato-oncology (PHO) departments or undergoing haematopoietic cell transplantation (HCT) between 2012 and 2023.

## 2. Materials and Methods

### 2.1. Design of the Study

In this multicentre nationwide study, we analysed the epidemiology, risk factors, clinical characteristics, and outcomes of staphylococcal infections in paediatric cancer patients or HCT recipients.

### 2.2. Patients and Data Collection

We performed a multicentre study, collecting data between 2012 and 2023 in Poland. The data included patients under 18 years of age (or under 21 years of age for HCT patients) receiving cancer therapy in 17 Polish paediatric haematology and oncology centres or undergoing HCT in 6 Polish paediatric centres. Data were collected continuously at 2-year intervals. Data collection was standardised across all participating centres using a unified electronic spreadsheet developed by the study coordinators. The dataset included predefined variables with consistent definitions and coding to ensure uniformity. All centres provided data based on this standardised template, with local data entry overseen by designated clinicians responsible for infection surveillance.

### 2.3. Definitions

Only microbiologically documented SI infections were evaluated. The infections were categorised into the following classifications: bloodstream, urinary tract, gastrointestinal, respiratory, and skin/soft tissue infections. The classification was based on culture results from sterile or non-sterile sites, depending on the clinical context and specimen type. Staphylococcal colonisations were not analysed in this study. Microbiological diagnostics were conducted in accredited hospital laboratories employing standardised methods. Pathogen identification and antimicrobial susceptibility testing were performed in accordance with the European Committee on Antimicrobial Susceptibility Testing (EUCAST) guidelines applicable at the time of analysis [9]. All laboratories participated in routine internal and external quality assurance programs to ensure the reliability and comparability of results across centres. Microbiologically documented infections were defined according to the definitions of the Infectious Diseases Working Party of the European Society of Blood and Marrow Transplantation [10]. Bloodstream infections, urinary tract infections, pneumonia, and wound infections were diagnosed by bacterial isolation from blood, urine, lower respiratory tract samples, and wound swabs, respectively, combined with the presence of clinical symptoms. Infections were analysed comparatively for patients from PHO centres and transplant centres.

The time to SI was defined as the time from diagnosis of malignancy to infection for PHO patients and the time from day of transplantation to infection for HCT patients. In a separately analysed subgroup of PHO patients who experienced relapse of malignancy, we used the date of relapse as the reference point for calculating the time to infection, as it marked the beginning of a new phase of oncological treatment and immunosuppression. Antibiotic therapy for SI was continued until microbiological clearance and resolution of infection symptoms.

Treatment-related mortality was defined as death within 30 days of completion of treatment for infection that was not due to recurrence or secondary malignancy. The infection outcome was considered positive if the patient survived the infection, or negative if the patient died due to the infection. In cases of recurrence and progression of the malignant neoplasm, it was considered the primary cause of death, regardless of concurrent infection. 

### 2.4. Antimicrobial Prophylaxis 

Antimicrobial prophylaxis was used routinely in all centres for HCT patients during the neutropenic phase and included cephalosporin or oral penicillin. Antifungal prophylaxis was consistently used in all centres in all HCT patients during the neutropenic phase or immunosuppressive therapy and included posaconazole or, rarely, other azoles. Most patients received cotrimoxazole for prevention of *Pneumocystis jirovecii* infections. In addition, environmental prophylaxis was administered in all centres. Each centre implemented a local policy of non-specific anti-infective prophylaxis.

### 2.5. Statistical Methods 

The cumulative incidence of infection was calculated using a competing risk analysis (Fine–Gray model), starting from the day of transplantation (HCT setting) or the day of diagnosis of malignancy (PHO setting). Infection-free survival was analysed using the Kaplan–Meier method, and differences between curves were compared using the log-rank test. Categorical variables were analysed using the chi-squared test, and continuous variables were compared using the Mann–Whitney U test.

To identify risk factors for the development of Staphylococcal infections, univariate logistic regression was performed. Clinically relevant variables or those statistically significant in univariate analysis were subsequently included in multivariate logistic regression models to determine independent predictors of infection. These variables were also included in the Fine–Gray competing risk model to assess their association with the cumulative incidence of infection.

Univariate and multivariate Cox regression models were applied to identify factors associated with infection-free survival and infection-related mortality. Additionally, univariate and multivariate logistic regression models were used to identify independent predictors of mortality. Variables with *p*-values < 0.1 in univariate analysis, along with clinically relevant variables, were included in multivariate models.

The following variables were included in the risk factor analysis of outcomes: primary diagnosis (acute leukaemia vs. other diagnoses), sex (female vs. male), age (<5 vs. >5 years), time to infection (<3 vs. >3 months), treatment (HCT vs. non-HCT), and duration of anti-SI therapy from the beginning of infection (>10 vs. ≤10 days). The independent predictor variable was survival from SI. Odds ratios (ORs), hazard ratios (HRs) and 95% confidence intervals (CIs) were calculated. *p* < 0.05 was considered statistically significant. Patients with missing data for a specific variable were excluded from analyses involving that variable but were included in all other applicable analyses.

## 3. Results

### 3.1. Demographics 

Over the 12-year study period, a total of 11,313 newly diagnosed paediatric patients were treated with anticancer therapy in 17 PHO centres, and 2039 children underwent HCT (allo-HCT = 1540; auto-HCT = 499) in six HCT centres.

### 3.2. Characteristics of Patients with Staphylococcus spp. Infections

Among the study cohort, 1725 patients were diagnosed with *Staphylococcus* spp. infections. PHO patients (n = 1433) were diagnosed with acute lymphoblastic leukaemia (ALL), acute myeloid leukaemia (AML), non-Hodgkin lymphoma (NHL), Hodgkin disease (HD), myelodysplastic syndrome (MDS), chronic myeloid leukaemia (CML), histiocytosis (LCH), central nervous system tumour (CNST), neuroblastoma (NBL), Wilms tumour (WT), Ewing sarcoma (ES), osteosarcoma (OS), rhabdomyosarcoma (RMS), germ cell tumour (GCT), and others. Between 2012 and 2013, a group of solid tumours (n = 63) emerged in the centres’ data, including CNST, WT, ES, OS, RMS, GCT, or other solid tumours. HCT (n = 292) patients underwent transplantation due to ALL, AML, NBL, MDS, NHL, HD, severe aplastic anaemia (SAA), primary immunodeficiency (PID), ES, or other diseases. Most patients were transplanted from a matched unrelated donor (66.8%) and all (except SAA patients) underwent myeloablative conditioning. The median age of PHO patients was 4.6 years (min–max, 0.01–17.9 years) and 8.1 years for HCT recipients (min–max, 0.2–21.0 years). The majority of patients in both groups were boys (58.0% in PHO and 68.2% in HCT) (Table 1).

PHO relapsed patients (n = 136) were diagnosed with ALL, AML, NHL, HD, CNST, RMS, NBL, WT, GCT, and other (n = 14). The median age at diagnosis was 7.2 years (min–max, 0.1–17.9 years). The median age at relapse was 9.2 years (min–max, 0.3–19.6 years). The majority of relapsed patients were boys (n = 77, 56.6%) (Table 2). 

### 3.3. Incidence of Infections 

**Figure 1:**
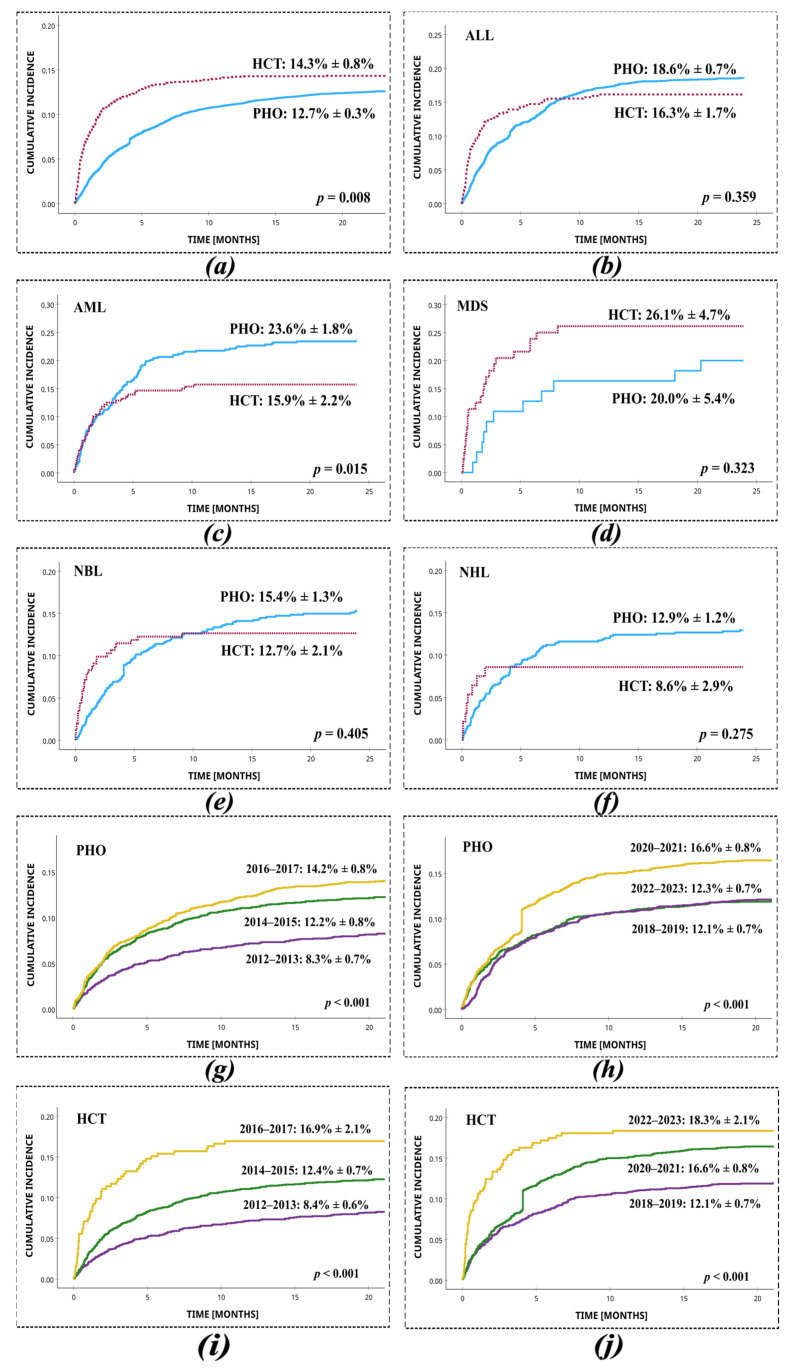
Cumulative incidence of infections with *Staphylococcus* spp. in HCT and PHO patients, with respect to the most common primary diseases and two-year time intervals. (**a**) Total, PHO vs. HCT; (**b**) ALL, PHO vs. HCT; (**c**) AML, PHO vs. HCT; (**d**) MDS, PHO vs. HCT; (**e**) NBL, PHO vs. HCT; (**f**) NHL, PHO vs. HCT; (**g**) PHO, 2012–2013 vs. 2014–2015 vs. 2016–2017; (**h**) PHO, 2018–2019 vs. 2020–2021 vs. 2022–2023; (**i**) HCT, 2012–2013 vs. 2014–2015 vs. 2016–2017; (**j**) HCT 2018–2019 vs. 2020–2021 vs. 2022–2023.

SI was diagnosed as a microbiologically documented infection in 1725 (1725/13352 = 12.9%) patients, including 1433 PHO and 292 HCT patients. Throughout the study period, the cumulative incidence of SI in PHO patients was 12.7% (95% CI: 12.4%–13.0%). In HCT patients, the cumulative incidence was 14.3% (95% CI: 13.5%–15.1%). The cumulative incidence of staphylococcal infections was significantly higher in HCT vs. PHO patients (*p* = 0.008) (Figure 1). A statistically significant difference of cumulative incidence between PHO and HCT patients was shown in the AML group (PHO: 23.6 ± 1.8% vs. HCT: 15.9 ± 2.2%; *p* = 0.015). The cumulative incidence of SI was analysed separately in PHO and HCT patients across consecutive two-year intervals between 2012 and 2023. In the PHO group, a significant variation in SI incidence was observed over time (*p* < 0.001), indicating a temporal trend in infection frequency within the population. Similarly, in the HCT group, SI incidence also varied significantly across the study period (*p* = 0.014), suggesting changing infection dynamics in this higher-risk cohort. At least two episodes of SI were reported in 224/1433 (15.6%) PHO patients and in 42/292 (14.4%) HCT patients. The highest number of episodes of SI during treatment of malignancy was five in the PHO patients and four in the HCT patients. The incidence of SI increased between 2012 and 2023. In PHO, the highest prevalence was reached in 2020–2021 (16.6% ± 0.8%), whereas in HCT, the highest prevalence of infection occurred in 2022–2023 (18.3% ± 2.1%).

Among all analysed cases, the most common source of staphylococcal infection in PHO and HCT patients was the bloodstream (83.6% and 89.0%, respectively). The incidences of microbiologically confirmed sources of infection are shown in Table 3. Staphylococcal colonisations were not analysed in the study.

In order to assess the proportion of staphylococcal infections originating from clinically sterile sites, we analysed the distribution of isolates obtained from the blood, cerebrospinal fluid, transplant material, and pleural/peritoneal fluid. Among PHO patients, infections caused by *S. aureus* originated from these sterile sites in 58.9% of cases, while for CoNS this proportion was 94.1%. In HCT patients, the corresponding values were 85.7% for *S. aureus* and 93.0% for CoNS.

The first episode of infection occurred significantly earlier in HCT recipients than in the patients who received conventional chemotherapy or radiotherapy. The median time to SI development was 3.6 months (min–max, 0.01–24.2 months) for PHO patients and 0.9 months (min–max, 0.01–18.8 months) for HCT patients. The incidence of SI was the highest among patients with AML in the PHO setting and with MDS in the HCT setting. The incidences of infections with *Staphylococcus* spp. in relation to the primary diagnosis are shown in Table 4. 

The most frequently isolated pathogen in PHO and HCT settings was *Staphylococcus epidermidis* (46.4% and 45.3%, respectively), followed by *S. aureus* and *S. hominis* in PHO and *S. haemolyticus* and *S. aureus* in HCT patients. Species identification was not possible for 64 (3.7%) CoNS isolates in PHO and for 4 (1.2%) CNS isolates in HCT (Table 5).

The competing risks regression analysis using the Fine–Gray model revealed that patients undergoing HCT had a significantly higher cumulative incidence of SI compared to PHO patients. Specifically, the subdistribution hazard ratio (SHR) for infection in the HCT group was 1.19 (95% CI: 1.04–1.35; *p* = 0.0096), indicating a 19% increased risk relative to the PHO group after accounting for competing risks. In univariate competing risks regression analysis, the diagnosis of acute leukaemia (ALL or AML) was significantly associated with the cumulative incidence of SI. Patients with acute leukaemia had a nearly twofold higher risk of developing infection compared to those with other malignancies (SHR = 1.92; 95% CI: 1.75–2.11; *p* < 0.001). Multivariate competing risks regression was subsequently conducted to identify independent predictors of infection incidence. The diagnosis of acute leukaemia remained a strong independent risk factor (HR = 1.91; 95% CI: 1.74–2.10; *p* < 0.001). In contrast, the increased risk observed for the HCT group compared to PHO patients was not statistically significant in the multivariate model (HR = 1.12; 95% CI: 0.98–1.27; *p* = 0.10).

Univariate risk factor analysis for the incidence of SI revealed that patients undergoing HCT had a slightly increased risk compared to non-HCT patients (OR = 1.2, 95% CI: 1.0–1.3, *p* = 0.04). Additionally, the presence of acute leukaemia (ALL or AML) was associated with a significantly higher risk of infection compared to other diagnoses (OR = 2.0, 95% CI: 1.8–2.2, *p* < 0.001). A more detailed analysis of infection risk could not be performed due to incomplete data on PHO and HCT patients without documented infection. In a multivariate logistic regression model that included both exposure to HCT and underlying malignancy, the presence of acute leukaemia was the only statistically significant risk factor for SI (OR = 2.0, 95% CI: 1.8–2.2, *p* < 0.001), while HCT had no impact on incidence (OR = 1.1, 95% CI: 0.9–1.3, *p* = 0.2).

Epidemiological data on infections in PHO relapsed patients (n = 136) were available for the period 2014–2023. The leading cause of infection was *S. epidermidis* with a frequency of 54.4%, followed by *S. haemolyticus* (21.3%), *S. hominis* (9.6%), and *S. aureus* (8.8%), along with further infections. The median time from the diagnosis of malignancy recurrence to the onset of infection was 4.7 months (min–max, 0.3–23.7 months).

### 3.4. Antibiotic Susceptibility 

Susceptibility to selected antibiotics was assessed for 1835 *Staphylococcus* spp. strains (1621 isolated from PHO and 214 from HCT), isolated from 1725 patients. The highest susceptibility rates were observed for linezolid (99.9% in PHO; 99.5% in HCT), vancomycin (99.3% in PHO; 100% in HCT), tetracycline (98.2% in PHO; 96.3% in HCT), and levofloxacin (97.8% in PHO; 95.8% in HCT). For the remaining antibiotics, susceptibility ranged from 52.3% to 96.8%. The in vitro antimicrobial susceptibilities of staphylococcal isolates are shown in Table 6.

### 3.5. Antibiotic Treatment 

In patients with microbiologically documented SI, targeted antibiotic treatment was applied. The median duration of antibiotic therapy was 10 days (min–max, 3–56 days) for PHO and 11 days (min–max, 3–49 days) for HCT patients. Among PHO relapsed patients, the median duration of SI treatment was 11 days (min–max, 3–87 days). Methicillin resistance of *Staphylococcus* spp. was well-characterised in 1787 PHO and 333 HCT infection cases. In PHO, 49/1787 (2.7%) methicillin-resistant *S. aureus* (MRSA) strains were identified, followed by 132/1787 (7.4%) methicillin-resistant *S. epidermidis* (MRSE) and 353/1787 (19.8%) MRCNS. In comparison, among HCT patients, 7/333 (2.1%) MRSA, 69/333 (20.7%) MRSE, and 53/333 (15.9%) MRCNS were detected. Furthermore, among relapsed PHO patients, methicillin-resistant *Staphylococcus* strains were detected, with MRSE being the most frequent (40/136, 29.4%), followed by MRCNS (26/136, 19.1%) and MRSA (3/136, 2.2%).

Complete treatment data were available for 1652 cases of infection in PHO and 325 cases in HCT patients. In the majority of the cases, vancomycin was the drug of choice. In the PHO setting, vancomycin was used as monotherapy in 374 cases (314/1652, 19.0%) and as a component of multidrug therapy for 532 cases (532/1652, 32.2%). In the HCT setting, vancomycin was used as monotherapy in 78 cases (78/325, 24.0%) and as a component of multidrug therapy for 90 cases (90/325, 27.7%). In the remaining cases, combinations of various antibiotic groups were used in the treatment of infection (e.g., amikacin, amoxicillin/clavulanic acid, cefepime, cefotaxime, ceftazidime, ceftriaxone, cefuroxime, ciprofloxacin, clindamycin, cloxacillin, gentamicin, imipenem, levofloxacin, linezolid, meropenem, metronidazole, piperacillin/tazobactam, rifampicin, teicoplanin, tigecycline, and trimethoprim/sulfamethoxazole), most often as combination therapy involving two to five antibiotics simultaneously. The most frequently used antibiotics in PHO patients were amikacin, piperacillin/tazobactam, and meropenem; however, in the HCT setting, teicoplanin, meropenem, and amikacin were most commonly administered (Table 7). 

Logistic regression analysis was conducted to compare the infection-related mortality between patients treated with vancomycin monotherapy and those treated with vancomycin in combination with other antibiotics. Among PHO patients, no significant difference in mortality was observed between the two treatment strategies (OR = 1.1, 95% CI: 0.3–4.2, *p* = 0.910). Similarly, among relapsed PHO patients, the type of vancomycin-based therapy was not associated with a difference in mortality risk (OR = 1.0, 95% CI: 0.5–2.4, *p* = 0.934). However, in the HCT subgroup, treatment with vancomycin in combination with other antibiotics was significantly associated with a higher risk of death compared to vancomycin monotherapy (OR = 7.9, 95% CI: 1.8–35.5, *p* = 0.007).

### 3.6. Outcome 

**Figure 2:**
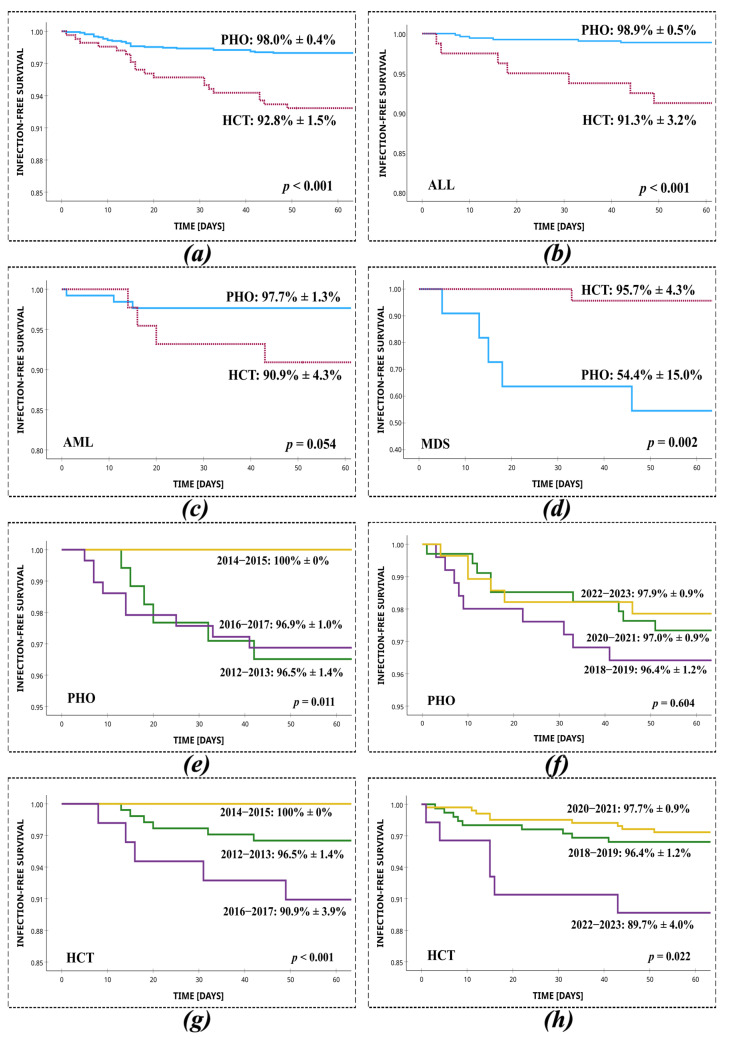
Survival from staphylococcal infection in PHO and HCT settings, with respect to the most common primary diseases and two-year time intervals. (**a**) Total, PHO vs. HCT; (**b**) ALL, PHO vs. HCT; (**c**) AML, PHO vs. HCT; (**d**) MDS, PHO vs. HCT; (**e**) PHO, 2012–2013 vs. 2014–2015 vs. 2016–2017; (**f**) PHO, 2018–2019 vs. 2020–2021 vs. 2022–2023; (**g**) HCT, 2012–2013 vs. 2014–2015 vs. 2016–2017; (**h**) HCT 2018–2019 vs. 2020–2021 vs. 2022–2023.

The overall successful outcome of treatment of SI was 98.0% in PHO and 92.8% in HCT patients (*p* < 0.001, log-rank). Among 1725 patients infected with *Staphylococcus* spp., the mortality associated with infection was 29/1433 (2.0%) in PHO patients and 20/292 (6.8%) in HCT patients. Deaths related to treatment failure of infection occurred after a mean of 17 days (95% CI, 12–21 days) in the PHO setting and in HCT patients, after a mean of 22 days (95% CI, 16–29 days). Thirty-day infection-free survival from SI in the PHO setting was significantly higher than among HCT patients (98.4% vs. 93.2%; *p* < 0.001, log-rank). Among non-surviving PHO patients, the most common underlying diagnoses were ALL (6/29, 20.7%), MDS (5/29, 17.2%), and AML (3/29, 10.3%). Similarly, among non-surviving HCT recipients, the most frequent prior diagnoses were ALL (7/20; 35.0%), AML (4/20; 20.0%), and chronic granulomatous disease (CGD; 2/20, 10.0%). The most common aetiologies of infection in non-surviving patients were *S. epidermidis* (PHO: 15/29, 51.7%; HCT: 8/20, 40.0%), *S. haemolyticus* (PHO: 4/29, 13.8%; HCT: 8/20, 40.0%), and *S. hominis* (PHO: 4/29, 13.8%, HCT: 2/20, 10.0%). At least two episodes of staphylococcal infection occurred in 5/29 (17.2%) of the non-surviving PHO patients and 3/20 (15.0%) of the non-surviving HCT patients. Regarding resistance mechanisms, in PHO patients, 12/29 (41.4%) of deaths were related to MRSE infection, 9/29 (31.0%) of deaths were related to MRCNS, and no deaths were observed following MRSA infection. Among HCT patients, 7/20 (35.0%) died due to MRSE infection, 6/20 (30.0%) due to MRCNS, and no deaths were observed following MRSA infection. A statistically significant survival difference between PHO and HCT patients was shown for the ALL (PHO: 98.9 ± 0.5% vs. HCT: 91.3 ± 3.2%; *p* < 0.001, log-rank) and MDS groups (HCT: 95.7 ± 4.3% vs. PHO: 54.4 ± 15.0%; *p* = 0.002, log-rank) (Figure 2). 

In univariate Kaplan–Meier survival analysis of infection-free survival (IFS), patients undergoing HCT had significantly shorter IFS compared to PHO patients (*p* < 0.001, log-rank). Age at infection and sex did not significantly influence IFS (*p* = 0.280 and *p* = 0.120, respectively; log-rank). Similarly, acute leukaemia diagnosis was not associated with IFS (*p* = 0.507, log-rank). In contrast, patients who developed infection more than 3 months after diagnosis demonstrated significantly longer IFS (*p* = 0.050, log-rank). Additionally, prolonged antibiotic therapy (>10 days) was associated with shorter IFS (*p* = 0.005, log-rank), potentially reflecting more severe or recurrent infectious episodes. 

In the univariate logistic regression analysis, undergoing HCT was significantly associated with increased mortality (OR = 3.6, 95% CI: 2.0–6.4, *p* < 0.001). Antibiotic therapy lasting more than 10 days also emerged as a significant mortality predictor (OR = 2.3, 95% CI: 1.3–4.1, *p* = 0.006). Other variables, such as sex (*p* = 0.123), age (*p* = 0.273), underlying acute leukaemia (*p* = 0.509), and time to infection (*p* = 0.053), did not reach statistical significance. In the multivariate model including all covariates, HCT remained an independent risk factor for a more than threefold increase in mortality risk (OR = 3.1, 95% CI: 1.6–5.9, *p* < 0.001). Male sex also emerged as statistically significant (OR = 1.8, 95% CI: 1.0–3.3, *p* = 0.039), suggesting a potential sex-related vulnerability. Prolonged antibiotic treatment remained a significant predictor of increased mortality (OR = 2.1, 95% CI: 1.1–3.8, *p* = 0.017), possibly reflecting more severe or complicated infections. Age, underlying diagnosis, and early-onset infection were not independently associated with mortality in the multivariate model. 

To complement the logistic regression and account for both the occurrence and timing of infection-related deaths, we performed a Cox proportional hazards regression analysis. This time-to-event approach provided additional insights into risk factors influencing mortality dynamics over time. In the univariate Cox analysis, undergoing HCT was significantly associated with a higher hazard of death compared to PHO patients (HR = 3.4, 95% CI: 1.9–6.1, *p* < 0.001). Antibiotic therapy longer than 10 days was also associated with increased mortality risk (HR = 2.2, 95% CI: 1.2–4.0, *p* = 0.007). Infections diagnosed within 3 months from initial diagnosis showed a trend toward poorer outcomes (HR = 0.6, 95% CI: 0.3–1.0, *p* = 0.053), although this did not reach conventional statistical significance. Sex, age, and the diagnosis of acute leukaemia were not significantly associated with mortality in univariate analysis. In the multivariate Cox model, both HCT (HR = 3.0, 95% CI: 1.6–5.6, *p* < 0.001) and prolonged antibiotic therapy (HR = 2.0, 95% CI: 1.1–3.6, *p* = 0.019) remained independent predictors of time to death, reinforcing the prognostic value of these factors beyond their association with mortality occurrence. Male sex was also identified as a significant predictor (HR = 1.8, 95% CI: 1.0–3.2, *p* = 0.038), despite showing no significance in univariate analysis, suggesting a possible interaction with other clinical variables. Notably, time to infection, patient age, and acute leukaemia were not independently associated with time to death in the adjusted model (Table 8). 

The survival from SI in PHO relapsed patients was 94.9% ± 1.9% (*p* = 0.019, log-rank), which was substantially lower than in PHO non-relapsed patients. Among 136 PHO relapsed patients infected with *Staphylococcus* spp., the mortality associated with this infection was 5.1% (7/136). The thirty-day survival from SI in the PHO relapsed patients was 96.3% ± 1.6%. Although the difference compared to PHO non-relapsed patients did not reach statistical significance (*p* = 0.079, log-rank), a trend toward reduced early survival was observed in the relapsed group. Deaths related to treatment failure in PHO infections occurred after a median of 10 days (95% CI, 5–34 days). Out of seven deaths, three cases occurred in ALL, two cases in CNST, one case in AML, and one case in NHL. There were two reported deaths linked to MRSE infection, and one fatality was attributed to MRSA infection. A univariate logistic regression analysis was performed to compare PHO patients with PHO relapsed patients regarding infection-related mortality within 30 days. The analysis revealed that staphylococcal infections in PHO relapsed patients were associated with increased mortality, but this association was not statistically significant in the 30-day mortality (*p* = 0.09). However, the odds ratio indicated a trend toward higher risk of infection-related death in relapsed patients, with an approximate 2.3-fold higher risk (OR = 2.3, 95% CI: 0.9–6.3).

## 4. Discussion

This study presents the results of the incidence and outcomes of *Staphylococcus* spp. infections in paediatric oncology and transplant centres participating in a nationwide multicentre long-term study on infectious complications. We analysed SI in two distinct patient populations at high risk of this complication due to prolonged hospitalisation, neutropenia, central venous catheter use, impaired immunity during chemotherapy cycles, or treatment of graft-versus-host disease (GvHD). The study focused on comparable parameters across diverse institutions, including university and general multidisciplinary centres.

Staphylococcal infections pose a significant clinical challenge and are among the most prevalent and impactful infectious complications in immunocompromised patients, particularly those undergoing treatment for malignancies or haematopoietic cell transplantation. The combination of a high prevalence, rising antimicrobial resistance, and the need for intensive, prolonged treatment contributes to the fact that staphylococcal infections remain a persistent burden for paediatric populations. This study provides a comprehensive analysis of the incidence and outcomes of SI in children who underwent haematopoietic cell transplantation and those who received conventional chemotherapy for the underlying disease. Comparative data on the epidemiology and outcomes of SI in HCT versus non-HCT paediatric patients are limited in the existing literature.

Our findings reveal that children undergoing HCT are particularly vulnerable to earlier and more frequent infections, often coinciding with periods of severe immunosuppression. In comparison, paediatric oncology patients generally experience infections later in the treatment course, reflecting differences in treatment protocols and immune recovery timelines. The study also underscores the high prevalence of methicillin-resistant strains, which pose additional challenges for treatment outcomes, especially in HCT patients [11]. Importantly, we observed a persistent and rising trend in the incidence of these infections, further emphasising the ongoing risk despite advances in cancer therapies. The data also indicate that prolonged treatment duration and undergoing HCT were independent risk factors associated with increased mortality in children with staphylococcal infections. A notable finding was the high frequency of bloodstream infections caused by *Staphylococcus* spp., particularly in patients with a central venous catheter (CVC). This emphasises the need for continued vigilance and targeted infection prevention strategies in high-risk paediatric populations.

The observed rate of infection, the prevalence of bloodstream infections, and the substantial mortality rate in the HCT group are comparable to those reported by other researchers. In a study conducted at a paediatric oncology centre, among 4198 positive samples representative of bloodstream infection, Gram-positive bacteria accounted for 24.2% of positive cultures, of which *Staphylococcus* spp. were isolated in 47.8% [12].

In our study, SIs were significantly more frequent and occurred earlier in HCT patients compared to those receiving conventional chemotherapy. The median time to infection was 0.9 months post-transplant in the HCT group, typically during profound myelosuppression and mucosal barrier damage. In contrast, infections in PHO patients occurred later, with a median of 3.6 months from diagnosis, often during the consolidation phase of chemotherapy. These findings highlight the increased vulnerability of HCT patients during the early post-transplant period and immune reconstitution [13,14]. It is essential to consider the different clinical starting points used for the median time to infection in each patient group, specifically PHO and HCT patients. For HCT patients, the time to infection was calculated from the day of transplantation, while for PHO patients, it was measured from the date of diagnosis of malignancy. This difference in definition, in addition to the timing of profound immunosuppression, may partially explain the shorter time to infection observed in the HCT group. Therefore, caution is warranted when directly comparing these values, as they reflect different phases of clinical care. Treatment outcomes and rates of infection-related treatment failure remained consistent over the study period. These findings underscore the importance of the issue in paediatric oncology. Although modern targeted therapies in oncology increase survival, they do not eliminate infection risk; instead, they often shift the pathogen spectrum [15].

*Staphylococcus aureus* and the CoNS, particularly *S. epidermidis* and *S. haemolyticus*, were the most frequently isolated pathogens in both the PHO and HCT settings. A similar dominance of the CoNS has been observed in studies of children with malignancy [16]. A retrospective study conducted at the University Hospital Centre Zagreb between 2017 and 2021 found that CoNS were the most frequently isolated pathogens, accounting for 49.6% of all isolates, with Gram-positive bacteria responsible for 69.1% of bloodstream infections in children with malignancies [17]. This confirms the predominant role of CoNS as the leading cause of bloodstream infections in paediatric oncology patients. Similarly, a 10-year study from a paediatric haematology–oncology unit found that CoNS represented 62.9% of all Gram-positive infections, with *S. epidermidis* being the most commonly isolated species [18]. In our study, the majority of infections were bacteraemias (more than 80%), which is coherent with the results of previous analyses [19,20,21]. Our analysis highlights that the majority of staphylococcal isolates originated from sterile clinical materials, particularly in CoNS cases. Although efforts were made to include only clinically significant infections, the retrospective design and reliance on culture-based diagnostics inherently carry a risk of misclassification, especially for isolates obtained from potentially contaminated sites. This limitation is particularly relevant for CoNS, which are common skin colonisers and may occasionally be misinterpreted as causative pathogens. Therefore, the reported incidence of non-bacteraemic CoNS infections from non-sterile sites should be interpreted with caution.

Dandoy et al. [22] showed that HCT patients had a higher risk of bloodstream infections compared to non-HCT patients. Bloodstream infections occurred in 31% of cases in patients after HCT, compared to 22% in patients treated with conventional chemotherapy. HCT patients had a higher mortality rate associated with bloodstream infections than non-HCT patients. Mortality due to bloodstream infections was 15% in HCT patients and 9% in non-HCT patients. 

According to the available literature, the most important risk factors for staphylococcal infections are the presence of a CVC, intensive chemotherapy, neutropenia, skin barrier dysfunction, and colonisation with resistant strains [4,23,24]. CVCs are the most common source of infection, and the risk of recurrent bacteraemia is particularly high with ported or tunnelled catheters [25,26]. The primary causative pathogens responsible for CVC-associated infections are those found in the skin, indicating the skin is a significant source of infection. Walker et al. [27] found that 85% of infections treated with antibiotic lock therapy (ALT) showed an initial cure rate, indicating the treatment’s high effectiveness. Patients undergoing HCT may also experience immunosuppression associated with GvHD treatment, which increases the risk of infection [22,28]. Retrospective studies show that infections occur more frequently in patients experiencing a relapse [18]. Acute leukaemia (ALL and AML) cases and MDS patients were particularly prone to infections, suggesting that deep and prolonged immunosuppression plays a significant part [29,30]. Research by Worku et al. [31] found that patients with haematological malignancies had a greater risk of developing bacterial infections than those with solid tumours. Our findings highlight the critical importance of underlying malignancy, particularly acute leukaemia, as the primary driver of both the risk and timing of staphylococcal infections in paediatric patients undergoing cancer treatment or HCT. While HCT was initially associated with increased infection risk, this effect was largely attributable to the predominance of high-risk diagnoses in this group. Clinically, this suggests that infection prevention and surveillance strategies should be prioritised and intensified in children with acute leukaemia, regardless of transplant status. 

Our study showed that survival after staphylococcal infection was significantly lower in HCT patients than in PHO. The mortality rate was 6.8% in HCT and 2.0% in PHO, confirming the results of other multicentre trials [22,32]. In a nationwide multicentre study involving PHO and HCT patients, infection-related mortality was found to be significantly higher in children undergoing haematopoietic cell transplantation compared to those receiving conventional anticancer therapy (5.9% vs. 1.4%, *p* < 0.0001), highlighting the increased vulnerability of transplant recipients to fatal infectious complications [1]. The majority of deaths were related to infections caused by MRSE and MRCNS [33,34,35]. Li et al. [36] demonstrated that in adult patients with malignancy, the 60-day mortality associated with MRSA bloodstream infections was 12.2%, and the 6-month mortality reached 43.2%, highlighting the serious impact of resistant staphylococcal infections in immunocompromised populations.

Analyses of risk factors associated with mortality showed that infection treatment for >10 days and HCT were independent risk factors for death. The observed association with male sex, though requiring further investigation, may point to underlying biological or clinical vulnerabilities. Previous research has shown that older age, male sex, ALL, high levels of C-reactive protein, and the presence of candidemia were linked to worse clinical outcomes [37]. Concerning the duration of treatment, the study by Lee et al. [38] demonstrated that patients with MRSA bacteraemia lasting ≥ 14 days experienced a mortality rate that was twice as high as that of patients with short-term bacteraemia (23.2% vs. 11.5%, *p* = 0.01). The prolonged antibiotic therapy may be an indicator of the severity of the course of the infection and potential life-threatening risk, highlighting the need to identify patients with persistent infection early and implement more aggressive therapeutic strategies. Furthermore, *S. aureus* bacteraemia and infection at an age under 1 year were found to be significant risk factors for high mortality in children [39]. 

MRSE and other MRCNS represent a major clinical challenge in paediatric oncology. The *mecA* gene typically mediates resistance by encoding a modified penicillin-binding protein (PBP2a) with a low affinity for β-lactam antibiotics, resulting in decreased effectiveness of this class of drugs and recurrent multidrug resistance [40]. In our study, MRCNS were the predominant pathogens in bloodstream infections. This is consistent with data from multiple centres. In a large cohort of paediatric oncology patients in Wuhan, *S. epidermidis* comprised 19.8% of blood culture isolates, with over 94% of these reported as resistant to penicillin G, while their susceptibility to teicoplanin, linezolid, and levofloxacin remained high [41]. In Jordan, CoNS constituted approximately half of all Gram-positive isolates in febrile neutropenia episodes, and all MRCNS remained susceptible to glycopeptides and linezolid [42]. Similarly, in a Polish single-centre study, CoNS made up 41–47% of all bloodstream isolates, and methicillin resistance among these strains ranged from 77% to 86% [43]. The clinical impact of MRSE/MRCNS is increased by their ability to form biofilms on central venous catheters and other medical equipment, leading to persistent bloodstream infections and treatment failure. A recent study from another Polish centre also confirmed CoNS as the dominant Gram-positive isolates, particularly in patients with long-term central lines [44]. Furthermore, emerging methicillin resistance phenotypes such as borderline oxacillin-resistant coagulase-negative Staphylococci and oxacillin-susceptible but mecA-positive coagulase-negative Staphylococci complicate detection and therapeutic decision-making. These phenotypes may not be accurately identified using conventional phenotypic testing alone, necessitating combined genotypic and phenotypic diagnostic strategies [45]. Infection control strategies, including meticulous catheter care, local resistance surveillance, and early pathogen-targeted therapy, are essential for improving outcomes in this high-risk population [46]. The persistent and widespread presence of MRSE/MRCNS in paediatric oncology patients highlights the need for continued antimicrobial stewardship and diagnostic improvement. 

Treatment of infections with resistant staphylococcal strains requires vancomycin, linezolid, teicoplanin, or a combination of these antibiotics. The effectiveness of therapy depends on the prompt implementation of targeted treatment that accounts for local resistance patterns [47,48]. However, in severe cases, such as sepsis during profound immunosuppression and mucosal barrier injury, even targeted therapy based on antibiograms may be insufficient.

Recent studies have raised concerns regarding the use of vancomycin monotherapy for serious staphylococcal infections, particularly in immunocompromised patients, due to its potential to promote resistance and suboptimal clinical outcomes. In a large retrospective cohort, vancomycin monotherapy was associated with a 52% treatment failure rate and higher mortality in patients with MIC ≥ 2 µg/mL [49]. Similarly, other reports demonstrated a more than fivefold increased risk of death with vancomycin monotherapy compared to combination regimens, most often including clindamycin [50]. According to the available data, vancomycin should be used with caution as monotherapy in children with MRSA infections. In cases of severe infections or when the MIC for vancomycin is ≥2 μg/mL, it is recommended to consider combination therapy or alternative drugs such as linezolid or daptomycin, depending on the location of the infection and the resistance profile of the pathogen [51]. Given the high proportion of monotherapy observed in our cohort, we aimed to assess its impact on infection-related mortality compared to combination treatment. However, in contrast to these findings, our study did not confirm a higher mortality rate among patients treated with vancomycin monotherapy. This result should be interpreted with caution, as it likely reflects confounding by indication. Patients who received combination therapy may have had more severe infections or additional complications, which prompted the use of broader-spectrum treatment. Therefore, the findings do not necessarily indicate that combination therapy is less effective. Further prospective evaluation is needed in specific paediatric subpopulations.

Alterations in microbiome composition can modulate skin colonisation by opportunistic pathogens, thereby increasing susceptibility to infection [52]. Chemotherapy and immunosuppressive therapy are known to disrupt the skin and gut microbiome, facilitating colonisation by opportunistic pathogens such as *Staphylococcus* spp., which can increase the risk of subsequent systemic infections. Colonisation of the nostrils and skin with *Staphylococcus aureus* (especially MRSA) is a well-recognised risk factor for invasive infections in patients after HCT [53]. Several studies have shown that decolonisation with mupirocin and chlorhexidine reduces the risk of infection and improves patient outcomes [54,55]. However, the increasing resistance of *S. aureus* to antiseptics such as chlorhexidine (mediated by the *qacA/B* gene) has been reported, potentially complicating infection control efforts in healthcare settings [26]. 

New strategies for identifying patients with a heightened risk of infection, involving models that consider the type of malignancy, transplant type, colonisation, and past infections, are currently being explored [56]. Recent diagnostic techniques, like whole-genome sequencing (WGS), facilitate the detection of hospital-acquired outbreaks and the identification of strains resistant to multiple drugs. WGS has emerged as a valuable tool for identifying clonal relationships in outbreaks of *Staphylococcus* spp., enabling precise detection of transmission pathways and informing infection control strategies. Studies have demonstrated that incorporating WGS into routine surveillance allows early identification of resistant strains and supports targeted interventions, especially in high-risk settings such as paediatric oncology and transplant units [57,58].

One of the most important aspects of our study is the inclusion of a large cohort of patients across multiple centres, which allows for a comprehensive analysis of the epidemiology and outcomes of SI in a diverse patient population. A key strength of the study is the direct comparison between two distinct patient groups: those undergoing HCT and those receiving conventional chemotherapy for malignancies. This comparison provides valuable data on differences in infection incidence, timing, and treatment outcomes between these high-risk groups, a subject that has been under-explored in the previous literature. Furthermore, the analysis of infection timing in relation to the intensity of immunosuppressive therapy provides detailed insight into how treatment modalities influence the course of infection.

The limitations of our study were the lack of complete clinical data on uninfected patients, the lack of colonisation data, the inability to analyse detailed in vitro resistance in some strains, the lack of data from the centres on relapsed patients during the specified years, and the retrospective character of the study. Additionally, we acknowledge that CoNS isolated from non-sterile sites pose an inherent risk of misclassification, and some infections included in the analysis may have been overestimated in terms of their clinical significance. Therefore, the results should be interpreted with caution, especially regarding infections caused by CoNS from non-sterile sources. Future prospective studies with stricter microbiological criteria and adjudication could help further clarify this issue. Nevertheless, the multicentre design and large sample size are the strengths of this study.

Clinicians should be aware that children undergoing HCT and those diagnosed with acute leukaemia are at particularly high risk of SI. The high incidence of bloodstream infections, increased methicillin resistance, and elevated infection-related mortality in HCT recipients underscore the importance of early recognition, tailored antimicrobial treatment, and stringent infection control measures. Close monitoring, especially in the early post-transplant period and in patients with central venous catheters or multiple comorbidities, is crucial for improved outcomes in this highly vulnerable group. Developing and implementing evidence-based strategies for antibiotic prophylaxis and targeted therapy are essential to reduce infection burden and improve survival.

## 5. Conclusions

Staphylococcal infections in children with malignancies and after haematopoietic cell transplantation remain a major therapeutic challenge. Our study provides important insights into the epidemiology, risk factors, and outcomes of SI in paediatric oncology and HCT patients. The findings highlight the importance of ongoing efforts to improve infection prevention, enhance diagnostic capabilities, and develop effective treatment strategies in order to confront the increasing threat posed by multidrug-resistant *Staphylococcus* species in vulnerable paediatric populations. Future studies should focus on the development of targeted therapies and more personalised approaches to managing infections in these high-risk groups.

## Figures and Tables

**Table 1:** Characteristics of paediatric patients with malignancies or HCT who developed *Staphylococcus* spp. infections.

PHO (n = 1433)		HCT (n = 292)	
Diagnosis		Diagnosis	
ALL	532 (37.1%)	ALL	81 (27.7%)
AML	128 (8.9%)	AML	44 (15.1%)
NHL	97 (6.8%)	MDS	23 (7.9%)
HD	43 (3.0%)	NHL	8 (2.7%)
MDS	11 (0.8%)	HD	4 (1.4%)
CML	1 (0.1%)	SAA	23 (7.9%)
LCH	12 (0.8%)	PID	17 (5.8%)
CNST	160 (11.2%)	NBL	32 (11.0%)
NBL	124 (8.7%)	ES	5 (1.7%)
WT	44 (3.1%)	Other *	55 (18.8%)
ES	34 (2.4%)		
OS	13 (0.9%)		
RMS	60 (4.2%)		
GCT	41 (2.9%)		
Solid tumours (2012/2013)	63 (4.4%)		
Other *	70 (4.9%)		
Age (yr/median/min–max)	4.6 (0.01–17.9)	Age (yr/median/min–max)	8.1 (0.2–21.0)
Gender		Gender	
Female	602 (42.0%)	Female	93 (31.8%)
Male	831 (58.0%)	Male	199 (68.2%)

n—number of included patients in the given category; PHO—paediatric haematology and oncology, HCT—haematopoietic cell transplantation, ALL—acute lymphoblastic leukaemia, AML—acute myeloblastic leukaemia, NHL, non-Hodgkin lymphoma, HD—Hodgkin disease, MDS—myelodysplastic syndrome, CML—chronic myeloid leukaemia, SAA—severe aplastic anaemia, LCH—Langerhans cell histiocytosis, PID—primary immunodeficiency, CNST—central nervous system tumour, NBL—neuroblastoma, WT—Wilms tumour, ES—Ewing sarcoma, OS—osteosarcoma, RMS—rhabdomyosarcoma, GCT—germ cell tumour. * The full list of underlying diseases grouped under the “Other” category is provided in the Supplementary Material (Table S1 for PHO patients and Table S2 for HCT patients).

**Table 2:** Characteristics of PHO relapsed patients and clinical sources of staphylococcal infections.

PHO Relapsed Patients (N = 136)		
Diagnosis		
ALL	57 (41.9%)	
AML	18 (13.2%)	
NHL	11 (8.1%)	
HD	3 (2.2%)	
NBL	15 (11.0%)	
RMS	7 (5.1%)	
WT	2 (1.5%)	
CNST	8 (5.9%)	
GCT	1 (0.7%)	
Other	14 (10.3%)	
Age at diagnosis (yr/median/min–max)	7.2 (0.1–17.9)	
Age at relapse (yr/median/min–max)	9.2 (0.3–19.6)	
Gender		
Female	59 (43.4%)	
Male	77 (56.6%)	
Source of infection		
	*S. aureus* (n = 12)	*CoNS* (n = 124)
Blood	7 (58.3%)	117 (94.4%)
Urine	-	3 (2.4%)
Injection site swab	1 (8.3%)	1 (0.8%)
Bronchoalveolar lavage	2 (16.7%)	-
Pharyngeal swab	1 (8.3%)	-
Sputum	-	1 (0.8%)
Cerebrospinal fluid	-	1 (0.8%)
Wound, pus	1 (0.8%)	-
Other *		1 (0.8%)

N—number of included PHO relapsed patients; n—number of *Staphylococcus* spp. isolates in the corresponding subgroup; PHO—paediatric haematology and oncology, ALL—acute lymphoblastic leukaemia, AML—acute myeloblastic leukaemia, NHL, non-Hodgkin lymphoma, HD—Hodgkin disease, NBL—neuroblastoma, RMS—rhabdomyosarcoma, WT—Wilms tumour, CNST—central nervous system tumour, GCT—germ cell tumour. * The full list of underlying diseases grouped under the “Other” category is provided in the Supplementary Material (Table S3 for PHO relapsed patients).

**Table 3:** Source of staphylococcal infections.

	PHO Patients (N = 1433)		HCT Patients (N = 292)	
	*S. aureus*	*CoNS*	*S. aureus*	*CoNS*
	(n = 314)	(n = 1119)	(n = 35)	(n = 257)
Blood	183 (58.3%)	1027 (91.8%)	30 (85.7%)	230 (89.5%)
Urine	5 (1.6%)	17 (1.5%)	2 (5.7%)	5 (1.9%)
Stool	5 (1.6%)	4 (0.4%)	-	1 (0.4%)
Pharyngeal swab	17 (5.4%)	2 (0.2%)	-	-
Urethral/foreskin/vaginal swab	1 (0.3%)	1 (0.1%)	-	1 (0.4%)
Transplant material	-	-	-	6 (2.3%)
Nasal swab	7 (2.2%)	-	-	-
Sputum	1 (0.3%)	-	-	-
Cerebrospinal fluid	2 (0.6%)	21 (1.9%)	-	2 (0.8%)
Wound, pus	54 (17.2%)	23 (2.1%)	1 (2.9%)	4 (1.6%)
Soft tissue	-	1 (0.1%)	-	1 (0.4%)
Skin swab	21 (6.7%)	13 (1.2%)	2 (5.7%)	5 (1.9%)
Oral cavity swab	2 (0.6%)	-	-	-
Sinus material	-	3 (0.3%)	-	-
Bronchoalveolar lavage	9 (2.9%)	-	-	1 (0.4%)
Conjunctival swab	5 (1.6%)	1 (0.1%)	-	-
Pleural/peritoneal fluid	-	4 (0.4%)	-	1 (0.4%)
Ear swab	2 (0.6%)	1 (0.1%)	-	-
Other	-	1 (0.1%)	-	-

N refers to the number of patients in each group; n refers to the number of *Staphylococcus* isolates. Percentages were calculated using n as the denominator in each subgroup. Percentages indicate the proportion of isolates obtained from each source.

**Table 4:** Incidences of infections with *Staphylococcus* spp. in relation to the primary diagnosis.

PHO Patients		HCT Patients	
Total	1433/11313 (12.7%)	Total	292/2039 (14.3%)
ALL	532/2866 (18.6%)	ALL	81/497 (16.3%)
AML	128/543 (23.6%)	AML	44/277 (15.9%)
NHL	97/750 (12.9%)	MDS	23/88 (26.1%)
HD	43/859 (5.0%)	NHL	8/93 (8.6%)
MDS	11/55 (20.0%)	HD	4/52 (7.7%)
CML	1/48 (2.1%)	SAA	23/208 (11.1%)
LCH	12/178 (6.7%)	PID	17/218 (7.8%)
CNST	160/1527 (10.5%)	NBL	32/253 (12.6%)
NBL	124/808 (15.3%)	ES	5/76 (6.6%)
WT	44/551 (8.0%)	Other *	55/277 (19.9%)
ES	34/173 (19.7%)		
OS	13/111 (11.7%)		
RMS	60/400 (15.0%)		
GCT	41/370 (11.1%)		
Solid tumours (2012/2013)	63/1012 (6.2%)		
Other *	70/1062 (6.6%)		

PHO—paediatric haematology and oncology, HCT—haematopoietic cell transplantation, ALL—acute lymphoblastic leukaemia, AML—acute myeloblastic leukaemia, NHL, non-Hodgkin lymphoma, HD—Hodgkin disease, MDS—myelodysplastic syndrome, CML—chronic myeloid leukaemia, SAA—severe aplastic anaemia, LCH—Langerhans cell histiocytosis, PID—primary immunodeficiency, CNST—central nervous system tumour, NBL—neuroblastoma, WT—Wilms tumour, ES—Ewing sarcoma, OS—osteosarcoma, RMS—rhabdomyosarcoma, GCT—germ cell tumour. * The full list of underlying diseases grouped under the “Other” category is provided in the Supplementary Material (Table S1 for PHO patients and Table S2 for HCT patients).

**Table 5:** Distribution of staphylococcal infections in all microbiologically confirmed cases.

	PHO Centres (n = 1786)	HCT Centres (n = 333)
*S. aureus*	374 (20.9%)	43 (12.9%)
*S.* *epidermidis*	828 (46.4%)	151 (45.3%)
*S.* *haemolyticus*	213 (11.9%)	79 (23.7%)
*S.* *hominis*	243 (13.6%)	42 (12.6%)
*S. warneri*	17 (1.0%)	6 (1.8%)
*S. cohnii*	2 (0.1%)	0 (0.0%)
*S. lentus*	7 (0.4%)	0 (0.0%)
*S. lugdunensis*	3 (0.2%)	0 (0.0%)
*S. xylosus*	1 (0.1%)	0 (0.0%)
*CoNS*	64 (3.7%)	4 (1.2%)
Other *	34 (1.9%)	8 (2.4%)

n refers to the total number of *Staphylococcus* spp. isolates identified in microbiologically confirmed infections. Percentages were calculated using n as the denominator in each group. * The category “Other” includes the following species: PHO group: *Staphylococcus capitis* (n = 19), *S. saprophyticus* (n = 7), *S. pasteuri* (n = 3), *S. simulans* (n = 2), and *Staphylococcus* spp. not further specified (n = 4); HCT group: *S. capitis* (n = 4), *S. simulans* (n = 1), *S. saprophyticus* (n = 1), and *Staphylococcus* spp. not further specified (n = 2).

**Table 6:** Susceptibilities to selected antibiotics of *Staphylococcus* spp. strains isolated from PHO and HCT patients.

Antibiotic	PHO (n = 1621)	HCT (n = 214)
Amikacin	1383 (85.3%)	166 (77.6%)
Amoxicillin/clavulanic acid	1085 (66.9%)	112 (52.3%)
Ampicillin	1068 (65.9%)	157 (73.4%)
Cefepime	1130 (69.7%)	121 (56.5%)
Cefotaxime	1123 (69.3%)	121 (56.5%)
Ceftriaxone	1130 (69.7%)	121 (56.5%)
Cefuroxime	1134 (70.0%)	121 (56.5%)
Cephazoline	1125 (69.4%)	121 (56.5%)
Ciprofloxacin	1483 (91.5%)	199 (93.0%)
Clindamycin	1308 (80.7%)	189 (88.3%)
Cloxacillin	1096 (67.6%)	115 (53.7%)
Erythromycin	1359 (83.8%)	146 (68.2%)
Gentamicin	1289 (79.5%)	125 (58.4%)
Imipenem	1108 (68.4%)	120 (56.1%)
Levofloxacin	1586 (97.8%)	205 (95.8%)
Linezolid	1619 (99.9%)	213 (99.5%)
Meropenem	1108 (68.4%)	120 (56.1%)
Oxacillin	1087 (67.1%)	112 (52.3%)
Piperacillin/tazobactam	1124 (69.3%)	122 (57.0%)
Teicoplanin	1547 (95.4%)	189 (88.3%)
Tetracycline	1592 (98.2%)	206 (96.3%)
Tobramycin	1569 (96.8%)	180 (84.1%)
Trimethoprim/sulfamethoxazole	1439 (88.8%)	176 (82.2%)
Vancomycin	1609 (99.3%)	214 (100%)

n—number of *Staphylococcus* spp. isolates included in the susceptibility analysis (1621 from PHO and 214 from HCT patients; total n = 1835). Percentages were calculated using the total number of isolates in each subgroup as the denominator, due to limitations in the availability of isolate-specific susceptibility data.

**Table 7:** Antimicrobials used in PHO and HCT patients with SI.

Antibiotic	PHO (n = 1652)	HCT (n = 325)
Amikacin	341 (20.6%)	60 (18.5%)
Amoxicillin/clavulanic acid	111 (6.7%)	10 (3.1%)
Cefepime	167 (10.1%)	30 (9.2%)
Cefotaxime	30 (1.8%)	0 (0.0%)
Ceftazidime	132 (8.0%)	7 (2.2%)
Ceftriaxone	75 (4.5%)	3 (0.9%)
Cefuroxime	44 (2.7%)	5 (1.5%)
Ciprofloxacin	60 (3.6%)	12 (3.7%)
Clindamycin	45 (2.7%)	1 (0.3%)
Cloxacillin	95 (5.8%)	8 (2.5%)
Gentamicin	10 (0.6%)	1 (0.3%)
Imipenem	21 (1.3%)	4 (1.2%)
Levofloxacin	6 (0.4%)	2 (0.6%)
Linezolid	103 (6.2%)	45 (13.8%)
Meropenem	314 (19.0%)	71 (21.8%)
Metronidazole	83 (5.0%)	1 (0.3%)
Piperacillin/tazobactam	321 (19.4%)	46 (14.2%)
Rifampicin	5 (0.3%)	2 (0.6%)
Teicoplanin	271 (16.4%)	91 (28.0%)
Tigecycline	0 (0.0%)	3 (0.9%)
Trimethoprim/sulfamethoxazole	6 (0.4%)	0 (0.0%)
Vancomycin	906 (54.8%)	168 (51.7%)

n—number of infection episodes with available antimicrobial treatment data. Percentages were calculated using n as the denominator in each group.

**Table 8:** Univariate and multivariate Cox regression analysis of risk factors for SI-related mortality.

	Univariate Cox Regression		Multivariate Cox Regression	
Risk Factor	HR (95% CI)	*p*	HR (95% CI)	*p*
HCT vs. PHO	3.4 (1.9–6.1)	<0.001	3.0 (1.6–5.6)	<0.001
Male vs. female	1.6 (0.9–2.7)	0.123	1.8 (1.0–3.2)	0.038
Age < 5 vs. >5 years	1.4 (0.8–2.4)	0.282	1.2 (0.7–2.1)	0.556
Acute leukaemia vs. other diagnoses	0.8 (0.4–1.5)	0.508	0.8 (0.4–1.4)	0.436
Time to infection < 3 vs. >3 months	0.6 (0.3–1.0)	0.053	0.8 (0.4–1.5)	0.497
Time of infection therapy >10 vs. <10 days	2.2 (1.2–4.0)	0.007	2.0 (1.1–3.6)	0.019

## Data Availability

The raw data supporting the conclusions of this article will be made available by the authors on reasonable request.

## Supplementary Materials

The following supporting information can be downloaded at: https://www.mdpi.com/article/10.3390/jcm14155200/s1, Table S1: Detailed list of diagnoses categorised as “Other” within the Paediatric Haemato-Oncology (PHO) patient group in Table 1 of the manuscript; Table S2. Detailed list of diagnoses categorised as “Other” within the Haematopoietic Cell Transplant (HCT) patient group in Table 1 of the manuscript; Table S3. Detailed list of diagnoses categorised as “Other” within the Relapsed Paediatric Haemato-oncology patient group in Table 2 of the manuscript.

## Abbreviations

The following abbreviations are used in this manuscript:

PHO	Paediatric haemato-oncology
HCT	Haematopoietic cell transplantation
EUCAST	European Committee on Antimicrobial Susceptibility Testing
SI	Staphylococcal infection
S.	*Staphylococcus*
MRSA	Methicillin-resistant *Staphylococcus aureus*
MRSE	Methicillin-resistant *Staphylococcus epidermidis*
MRCNS	Methicillin-resistant coagulase-negative *Staphylococcus*
CoNS	Coagulase-negative *Staphylococcus*
OR	Odds ratio
HR	Hazard ratio
CI	Confidence interval
ALL	Acute lymphoblastic leukaemia
AML	Acute myeloid leukaemia
NHL	Non-Hodgkin lymphoma
HD	Hodgkin disease
MDS	Myelodysplastic syndrome
CML	Chronic myeloid leukaemia
LCH	Langerhans cell histiocytosis
CNST	Central nervous system tumour
NBL	Neuroblastoma
WT	Wilms tumour
ES	Ewing sarcoma
OS	Osteosarcoma
RMS	Rhabdomyosarcoma
GCT	Germ cell tumour
SAA	Severe aplastic anaemia
PID	Primary immunodeficiency
SHR	Subdistribution hazard ratio
CVC	Central venous catheter
ALT	Antibiotic lock therapy
GvHD	Graft-versus-host disease
PBP2a	Penicillin-binding protein 2a
WGS	Whole genome sequencing
