# Supplementary material for: Infections with Staphylococcus spp. in Children Undergoing Anticancer Therapy or Haematopoietic Cell Transplantation: A Nationwide Multicentre Study
Source: J Clin Med. 2025 Jul 22;14(15):5200. doi: 10.3390/jcm14155200 (PMC12346928; doi:10.3390/jcm14155200)
Supplement: Supplementary file 1 [file jcm-14-05200-s001.zip › jcm-3735515-supplementary.pdf]

Supplementary material

Table S1. Detailed list of diagnoses categorised as "Other" within the Paediatric Haemato-Oncology (PHO) patient group in Table 1 of the manuscript

**Disease name**	**Number of patients**
Severe aplastic anaemia	11
Hemophagocytic lymphohistiocytosis	7
Mixed phenotype acute leukaemia / Acute bilineage leukaemia	6
Methicillin-resistant peripheral nerve sheath tumour / Primitive neuroectodermal tumour	5
Primary immunodeficiency	5
Diffuse large B-cell lymphoma	5
Retinoblastoma	5
Mastocytosis	4
Juvenile myelomonocytic leukaemia	3
Chronic granulomatous disease	2
Pompe disease	2
Juvenile xanthogranuloma	2
Myelofibrosis	2
Systemic amyloidosis	2
Chronic myeloid leukaemia	1
Diamond-Blackfan anaemia	1
X-linked adrenoleukodystrophy	1
Medullary thyroid carcinoma	1
Chronic lymphocytic leukaemia	1
Mesenchymal hamartoma of the liver	1
Myeloproliferative neoplasm	1
Rosai-Dorfman disease	1
Clear cell sarcoma	1
Total	70

Table S2. Detailed list of diagnoses categorised as "Other" within the Haematopoietic Cell Transplant (HCT) patient group in Table 1 of the manuscript

**Disease Name**	**Number of Patients**
Fanconi anaemia	8
Hemophagocytic lymphohistiocytosis	7
Juvenile myelomonocytic leukaemia	7
Chronic granulomatous disease	4
X-linked adrenoleukodystrophy	4
Diamond-Blackfan anaemia	4
Acute bilineal leukaemia / Mixed phenotype acute leukaemia	3
Germ cell tumours	3
Metachromatic leukodystrophy	2
Congenital neutropenia	2
Malignant peripheral nerve sheath tumour / Primitive neuroectodermal tumour	2
Osteopetrosis	2
Chronic myeloid leukaemia	1
Pompe disease	1
Common variable immunodeficiency	1
Central nervous system tumours	1
Total	55

Table S3. Detailed list of diagnoses categorised as "Other" within the Relapsed Paediatric Haemato-oncology patient group in Table 2 of the manuscript

**Disease**	**Number of Patients**
Myelodysplastic syndrome	3
Desmoplastic small round cell tumour	2
Liver tumour	2
Osteosarcoma	2
Ewing sarcoma (ES)	2
Acute bilineal leukaemia / Mixed phenotype acute leukaemia	1

Disease	Number of Patients
Juvenile myelomonocytic leukaemia	1
Total	13
